# Carbon–hydrogen (C–H) bond activation at Pd^IV^: a Frontier in C–H functionalization catalysis

**DOI:** 10.1039/c4sc02591a

**Published:** 2014-09-29

**Authors:** Joseph J. Topczewski, Melanie S. Sanford

**Affiliations:** a Department of Chemistry , University of Michigan , Ann Arbor , MI 48108 , USA . Email: mssanfor@umich.edu ; Fax: +1 734 647 4865 ; Tel: +1 734 615 0451

## Abstract

The direct functionalization of carbon–hydrogen (C–H) bonds has emerged as a versatile strategy for the synthesis and derivatization of organic molecules. Among the methods for C–H bond activation, catalytic processes that utilize a Pd^II^/Pd^IV^ redox cycle are increasingly common. The C–H activation step in most of these catalytic cycles is thought to occur at a Pd^II^ centre. However, a number of recent reports have suggested the feasibility of C–H cleavage occurring at Pd^IV^ complexes. Importantly, these latter processes often result in complementary reactivity and selectivity relative to analogous transformations at Pd^II^. This mini review highlights proposed examples of C–H activation at Pd^IV^ centres. Applications of this transformation in catalysis as well as mechanistic details obtained from stoichiometric model studies are discussed. Furthermore, challenges and future perspectives for the field are reviewed.

## Abstract 2

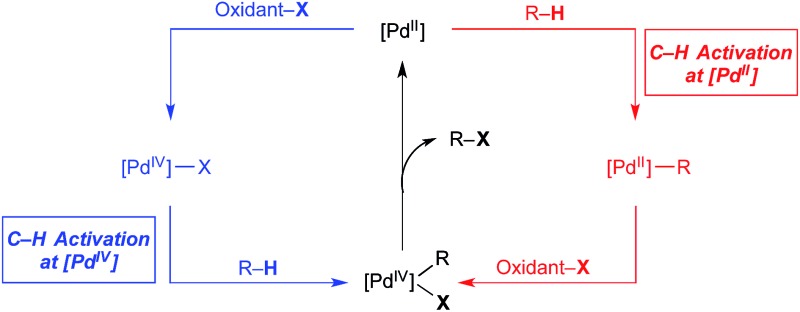
The direct functionalization of carbon–hydrogen (C–H) bonds has emerged as a versatile strategy for the synthesis and derivatization of organic molecules.

## Introduction

**Fig. 1:**
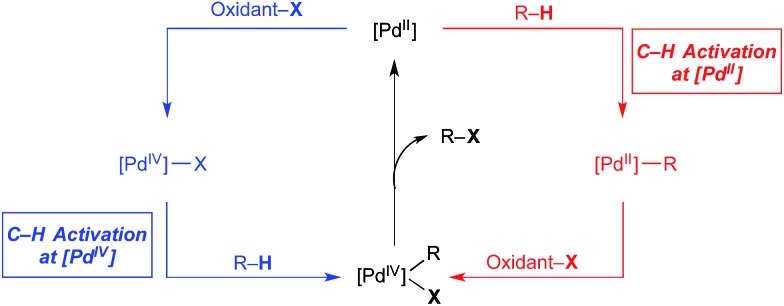
Examples of catalytic cycles involving C–H activation at Pd^II^ (red) *versus* Pd^IV^ (blue).

Over the past 15 years, catalytic C–H bond functionalisation has emerged as a rich and highly active field of research.^1^ C–H functionalisation reactions proceeding *via* Pd^II/IV^ catalytic cycles are particularly prevalent due to their operational simplicity, wide scope, excellent functional group tolerance, and opportunities to access both C–C and C-heteroatom bond construction.^2^ Pd^II/IV^-catalysed C–H functionalization reactions are generally proposed to proceed *via* catalytic cycles exemplified by that shown in red in Fig. 1. This involves three elementary steps: C–H activation at Pd^II^, 2*e* ^–^ oxidation to Pd^IV^ (or a Pd^III^ dimer)^3^ with an appropriate stoichiometric oxidant (oxidant-X), and finally C–X bond-forming reductive elimination from the high valent palladium centre to release the product.

Extensive research has established that the steps occur in this order for the vast majority of Pd^II/IV^-catalysed C–H functionalisations.^2^ However, several recent reports have suggested that C–H cleavage can also occur at Pd^IV^ centres (Fig. 1, blue) and, further, that this process may be governed by different selectivity and reactivity principles than analogous transformations at Pd^II^. This offers the exciting possibility for alternative catalytic cycles for Pd-catalysed C–H functionalisation, involving, for example, oxidation of Pd^II^ to Pd^IV^, C–H bond activation at Pd^IV^, and reductive elimination to release the product and regenerate the Pd^II^ catalyst. Notably, in both cycles in Fig. 1, additional and/or alternative steps are possible; however, the key distinguishing feature of the blue cycle, discussed herein, is that at least one C–H activation event occurs at Pd^IV^. This mini review summarizes examples where arene C–H activation at a Pd^IV^ centre is proposed in both catalytic transformations and in stoichiometric model systems.^4^ For some of these systems, clear experimental evidence demonstrates C–H activation at Pd^IV^ while for others, the role of C–H activation at Pd^IV^ is strongly suspected. Both synthetically useful catalytic cycles and mechanistic details are presented and discussed.^5^

## C–H activation at Pd^IV^

**Fig. 2:**
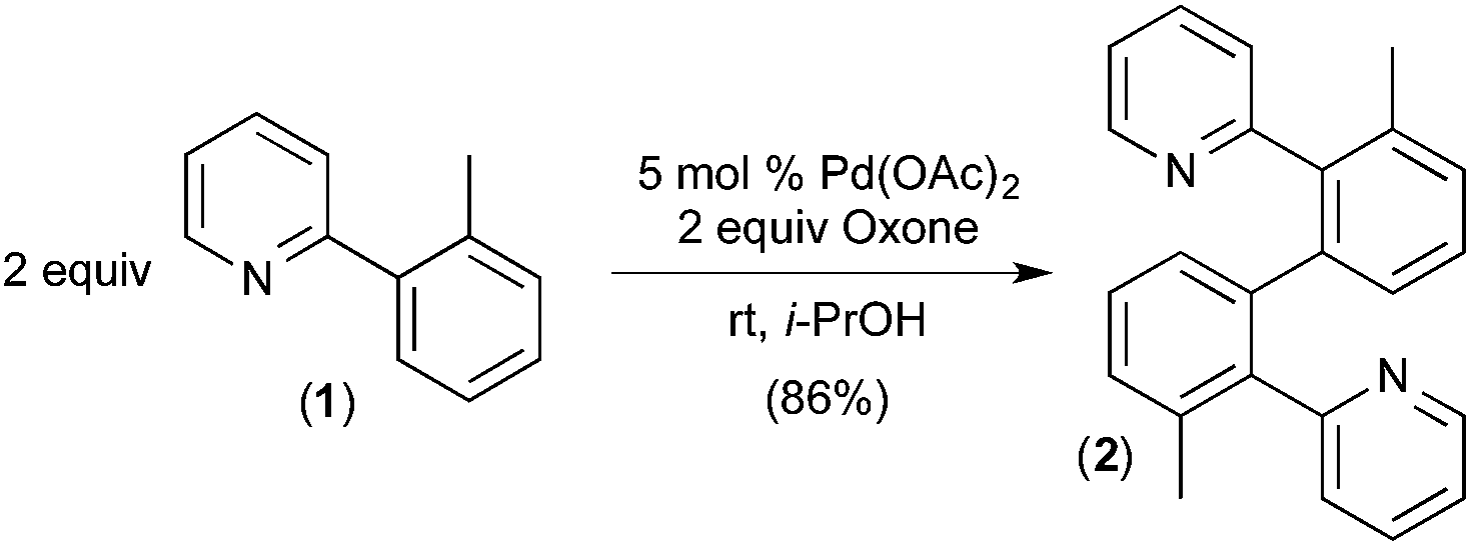
Oxidative coupling of 2-arylpyridine derivatives *via* proposed C–H activation at Pd^IV^.

To the best of our knowledge, the first report implicating a C–H activation reaction at Pd^IV^ involved the dimerization of 2-aryl pyridines.^6^ In this system, Pd(OAc)_2_ catalyses the C–H/C–H oxidative coupling of a variety of substituted 2-aryl pyridines at room temperature using Oxone as the terminal oxidant. A representative example is the conversion of 2 equiv. of **1** into **2** (Fig. 2).

**Fig. 3:**
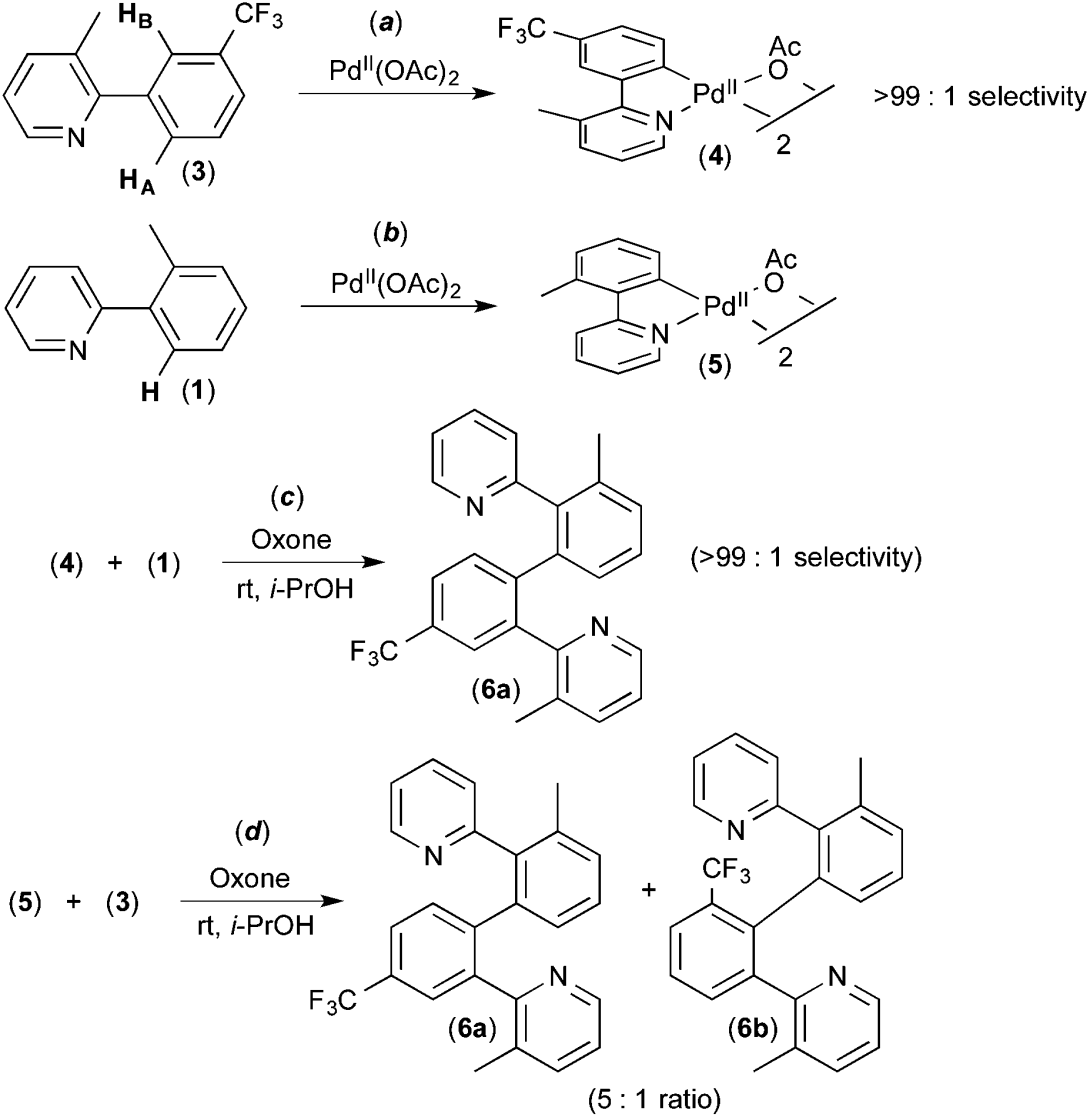
Experiments implicating two different C–H activation steps with different selectivities in activation of substrate **3**.

Several experiments were conducted that suggest that this transformation involves two discrete C–H activation steps that have very different selectivities. For example, the unsymmetrically-substituted substrate **3** undergoes stoichiometric cyclometalation with Pd^II^(OAc)_2_ to afford a single isomeric product **4** *via* selective cleavage of C–H_A_ (Fig. 3a). When this complex is subjected to Oxone and substrate **1** under the standard conditions, a single isomer of the coupled product is formed (**6a**, Fig. 3c). In contrast, when the sequence is reversed (*i.e.*, **1** is first cyclometalated at Pd^II^ to form **5** (Fig. 3b), and this intermediate is subjected to analogous conditions with substrate **3**), a 5 : 1 mixture of the isomeric products **6a** and **6b** is produced (Fig. 3d). These results implicate two different C–H activation steps with different selectivities: (i) the initial cyclometalation of **3** at Pd^II^(OAc)_2_ (>99 : 1 selectivity for activation of H_A_) and (ii) a subsequent C–H activation of **3** (5 : 1 selectivity for H_A_ *versus* H_B_).

**Fig. 4:**
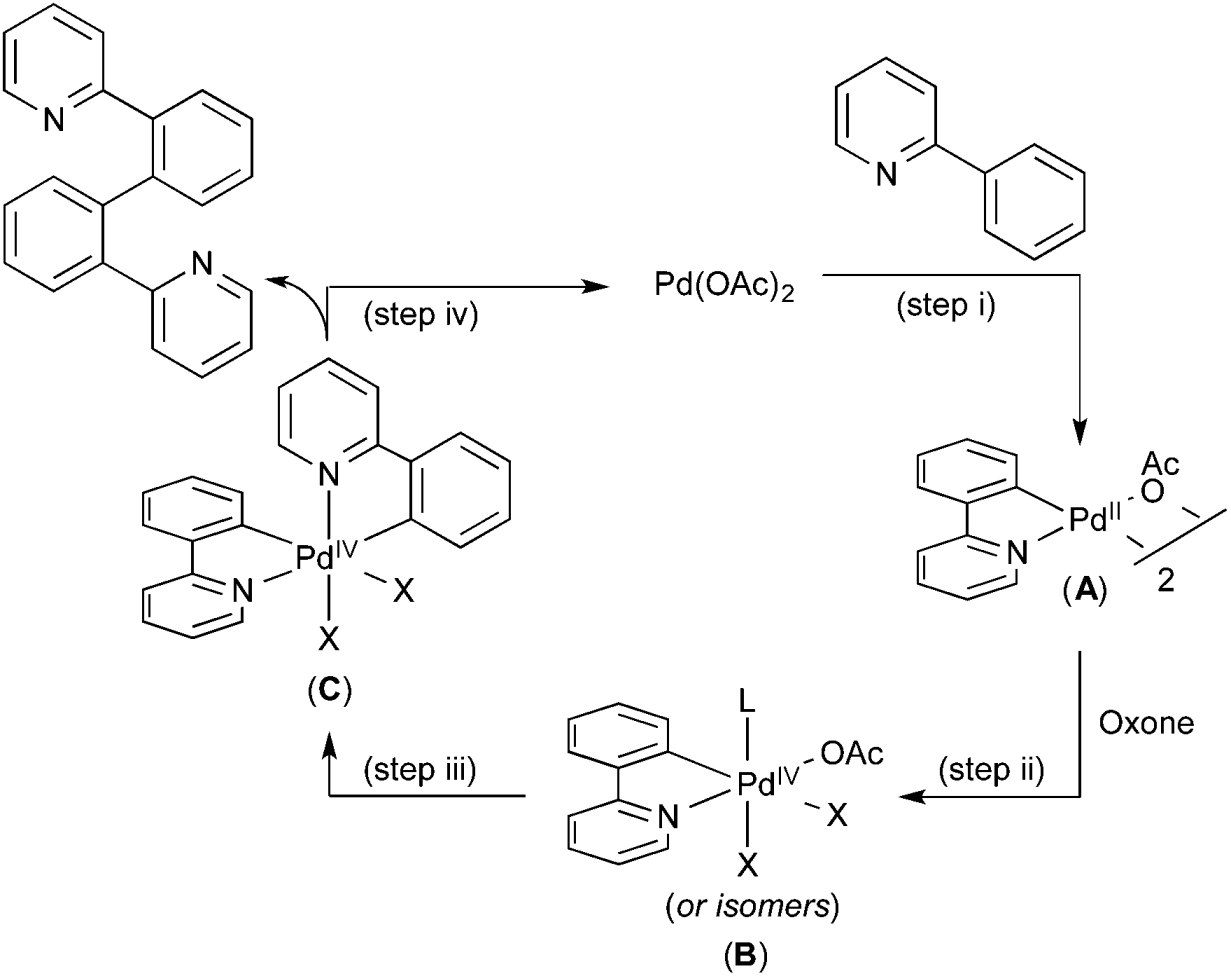
Proposed mechanism for Pd-catalysed oxidative coupling of 2-aryl pyridines.

A variety of additional experiments, including cross-over studies and reactivity studies of possible intermediates, implicated the mechanism shown in Fig. 4. Here, an initial C–H activation at Pd^II^ (step (i)), is followed by oxidation of the resulting palladacycle intermediate A with Oxone to yield Pd^IV^ species B (step (ii)). The second C–H activation then occurs at this Pd^IV^ intermediate to yield C (step (iii)), which undergoes C–C bond-forming reductive elimination to complete the catalytic cycle (step (iv)).

## Synthetic applications of C–H activation at Pd^IV^

**Fig. 5:**
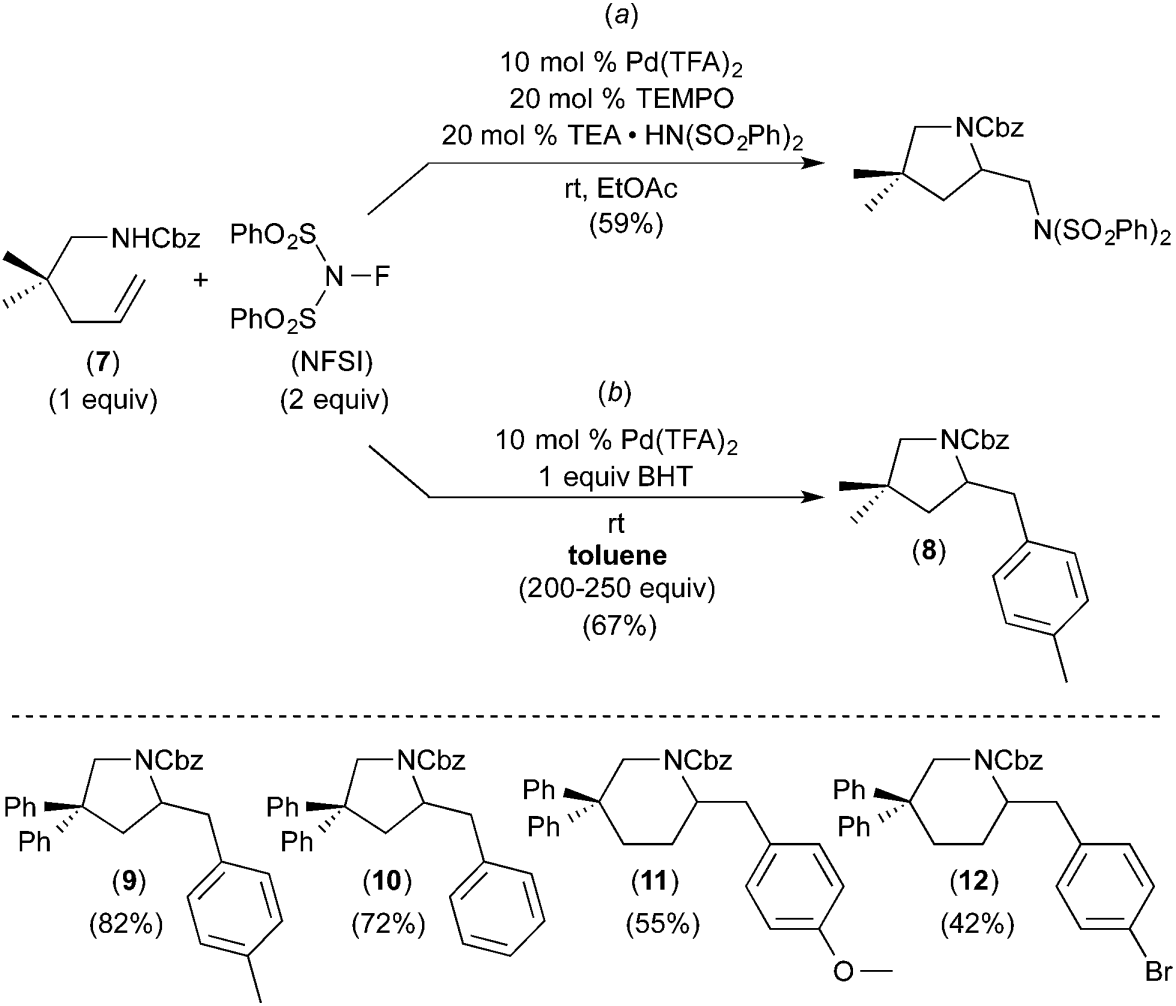
Alkene aminoarylation *via* proposed C–H activation at Pd^IV^.

Subsequent work has taken advantage of proposed arene C–H activation reactions at Pd^IV^ to achieve synthetically useful catalytic transformations. In one elegant example, Michael demonstrated the Pd^II/IV^-catalysed aminoarylation of terminal olefins with NFSI as the oxidant (Fig. 5).^7^ This reaction was discovered during an investigation of the Pd-catalysed diamination of **7** (Fig. 5a). When the solvent for this transformation was changed from EtOAc to toluene, the aminoarylation product **8** was formed *via* toluene C–H activation. A variety of substituted arenes can also be used in this transformation, with substituents including Br, CH_3_, and CH_3_O. Furthermore, mono-substituted arenes react with extremely high selectivity at the *para* position (*c.f.*, products **9–12** of Fig. 5). This high selectivity is in marked contrast to most other Pd-catalysed functionalisations of mono-substituted arenes, which typically form mixtures of isomeric products.^8^ Additionally, Michael's aminoarylations proceed efficiently at room temperature, which is significantly milder than most Pd^II^-catalysed C–H functionalizations of simple arenes. The authors propose that arene C–H activation occurs at a Pd^IV^ centre and that this feature is responsible for the unusually high selectivity and reactivity.

A number of experiments were conducted to gain further insights into the mechanism of this process. First, the electronic requirements of the C–H activation step were investigated using competition experiments between benzene and other arenes. These studies showed that benzene reacts faster (by a factor of ∼2.5) than both anisole and bromobenzene.

**Fig. 6:**
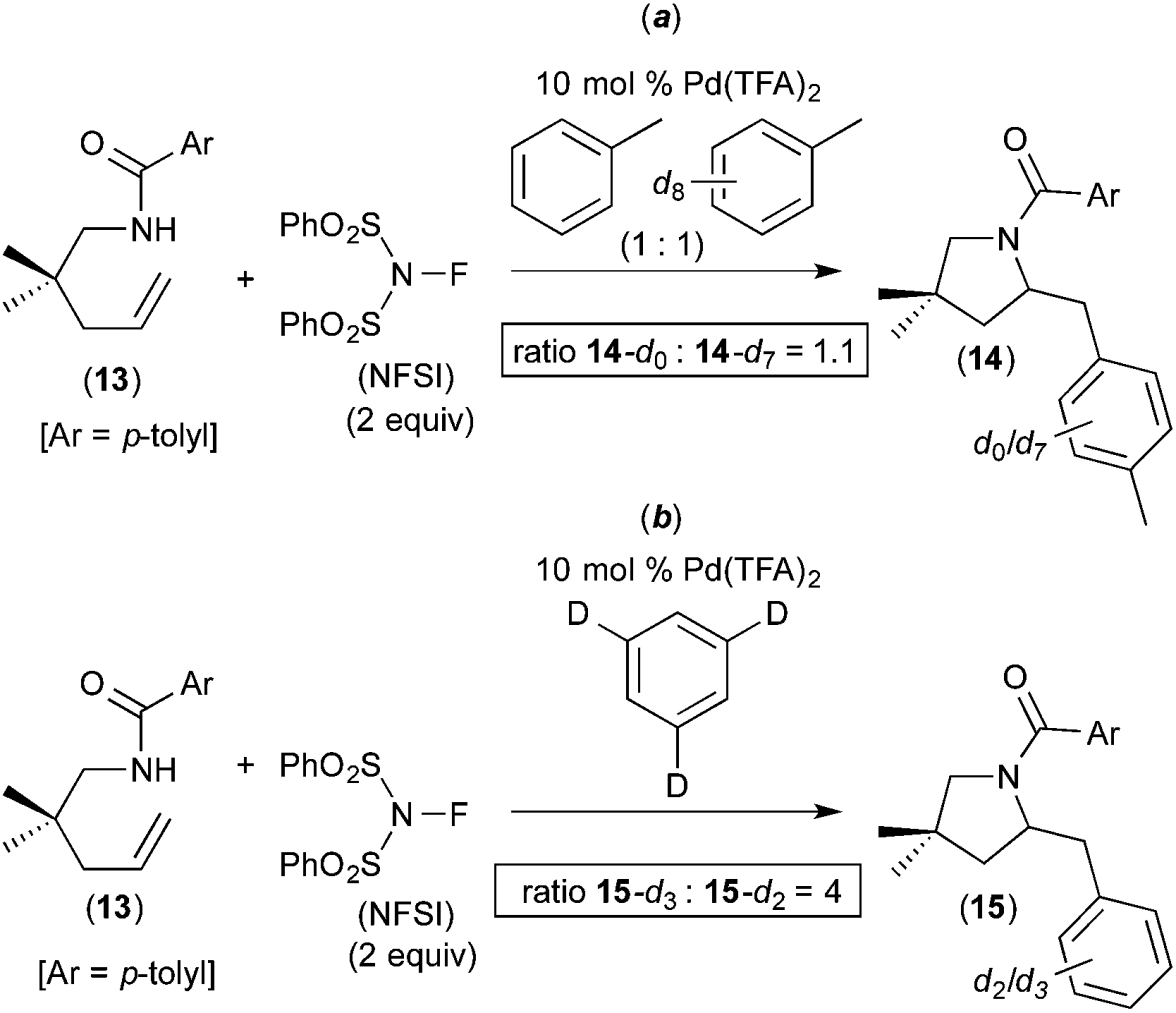
Kinetic isotope effect studies of alkene aminoarylation reaction.

**Fig. 7:**
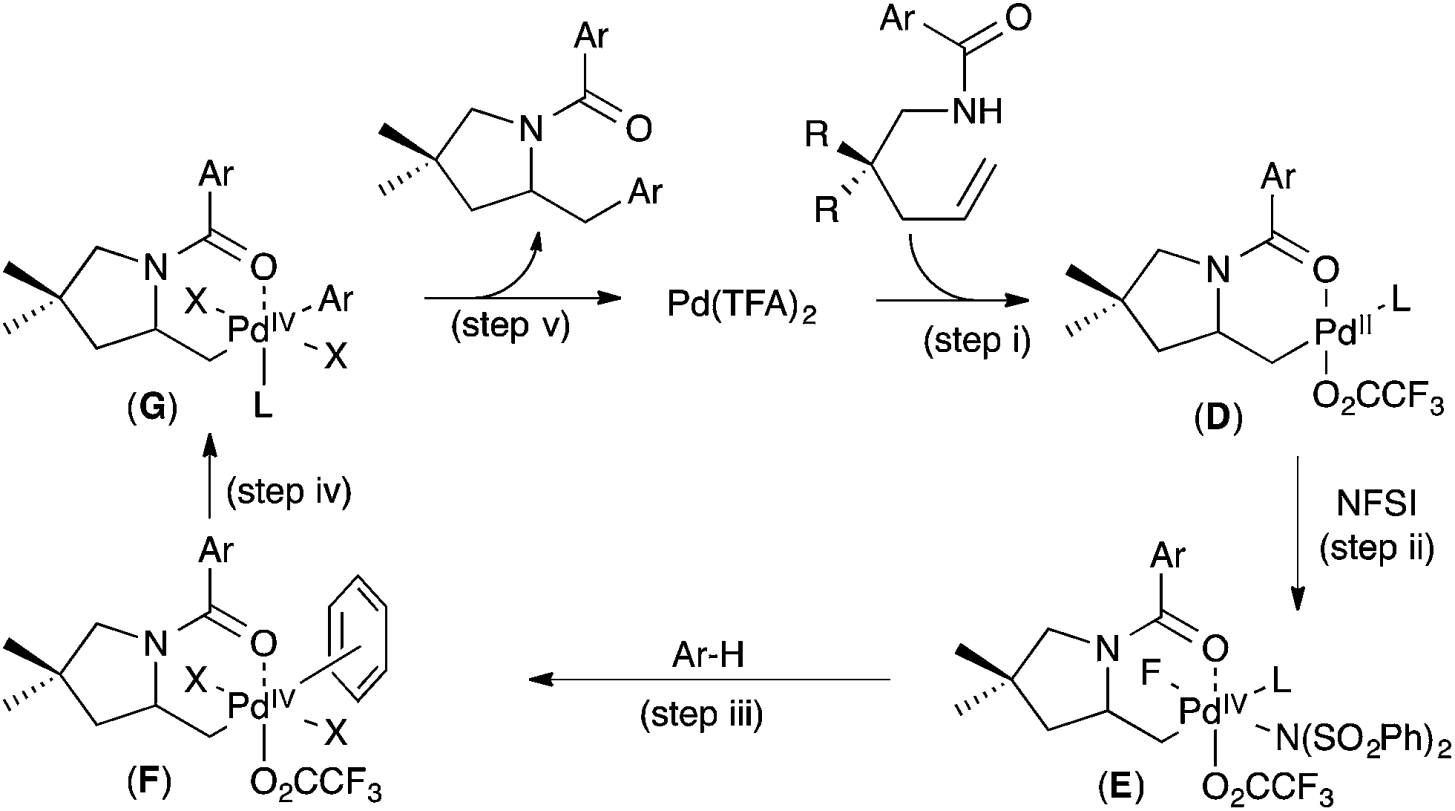
Proposed mechanism for aminoarylation.

A competition between toluene and toluene-*d*_8_ showed an intermolecular H/D competition isotope effect of 1.1 (**14**-*d*_0_/**14**-*d*_7_ = 1.1, Fig. 6a). In contrast, the use of 1,3,5-trideuterobenzene as the substrate resulted in a much larger intramolecular H/D competition isotope effect of 4 (**15**-*d*_3_/**15**-*d*_2_ = 4, Fig. 6b). In combination, these results implicate a 2-step C–H activation process, in which the two different steps occur with distinct selectivities. As shown in Fig. 7, the authors propose that the two steps are π-coordination of the arene to the Pd^IV^ centre (which determines the intermolecular isotope effect, step (iii) in Fig. 7) followed by C–H cleavage of the π-coordinated substrate (which dictates the intramolecular isotope effect, step (iv) in Fig. 7). Notably, C–H activation reactions at Pd^II^ centres generally show much higher intermolecular competition isotope effects (typically ranging from 2 to 6).^9^

On the basis of these (and additional) studies, a full catalytic cycle was proposed. As shown in Fig. 7, the cycle begins with intramolecular *anti*-aminopalladation to produce alkyl Pd^II^ intermediate D (step (i)). D then undergoes oxidation with NFSI to produce Pd^IV^ intermediate E (step (ii)) π-coordination of the arene substrate to E to generate F (step (iii)) is followed by C–H cleavage (step (iv)) to afford aryl alkyl Pd^IV^ complex G. Finally, C–C bond-forming reductive elimination (step (v)) closes the catalytic cycle.

**Fig. 8:**
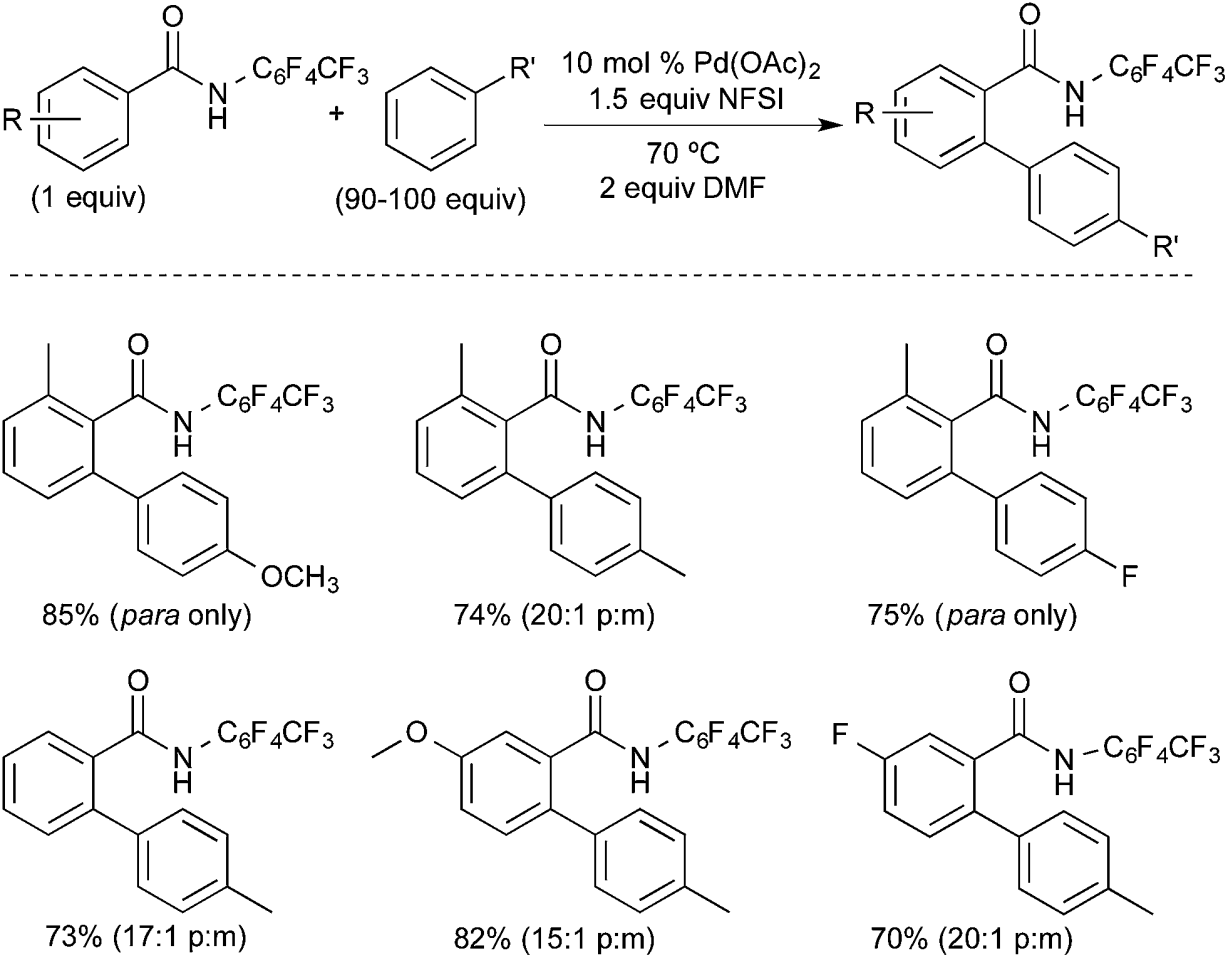
Arene C–H oxidative coupling involving proposed C–H activation at Pd^IV^.

The Yu group reported a related Pd^II/IV^-catalysed C–H functionalization reaction involving the oxidative coupling of perfluorobenzamides with simple arenes using NFSI as the oxidant (Fig. 8).^10^ Similar to Michael's work, this transformation proceeds with very high *para* selectivity, ranging from 12 : 1 with a bromo- or ethyl-substituent to “*para* only,” with methoxy- or fluoro-substituents. The authors rationalize this unusually high selectivity based on a mechanism involving two sequential C–H activation events: ligand-directed C–H activation at Pd^II^ followed by arene C–H activation at Pd^IV^. They propose that the weakly coordinating perfluorobenzamide directs an initial C–H activation at Pd^II^. The NFSI then oxidizes this palladacyclic intermediate to a Pd^IV^ fluoride complex, which promotes *para*-selective arene C–H activation.^11^

Several studies were conducted to shed further light on the mechanism of this transformation. While substantial yields of oxidative coupled products were obtained with a number of different oxidants (*e.g.*, Selectfluor, *N*-fluoropyridinium K_2_S_2_O_8_), only F^+^ oxidants afforded high levels of *para*-selectivity. This led the authors to propose that the presence of a fluoride ligand on the Pd^IV^ center is crucial for achieving *para*-selective C–H activation.

**Fig. 9:**
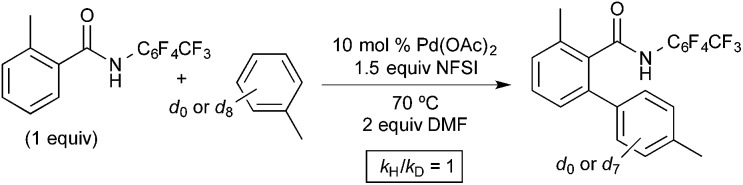
Isotope effect study for arene C–H oxidative coupling.

As shown in Fig. 9, an isotope effect study revealed that the initial reaction rate is identical with toluene and toluene-*d*_8_ as the arene substrate (*k*_H_/*k*_D_ = 1). This result suggests that the C–H activation at Pd^IV^ is not the slow step of the catalytic cycle. Unlike the Michael system, no competition or intramolecular isotope effect studies were reported in this system, so the possible role of π-coordination cannot be assessed from this report.^10^

**Fig. 10:**
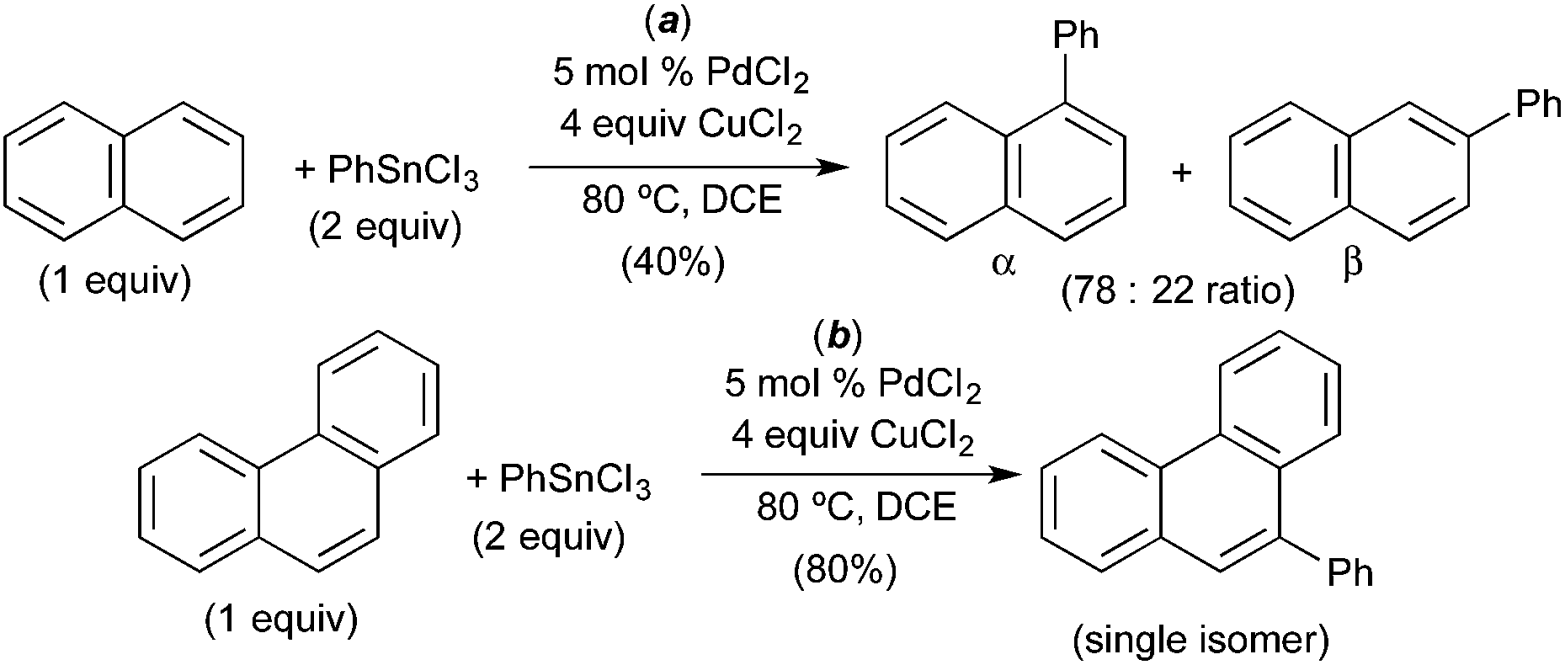
C–H arylation of arenes with PhSnCl_3_ *via* proposed C–H activation at Pd^IV^.

A number of groups have used naphthalene as a substrate in C–H arylation reactions that are believed to proceed *via* C–H activation at Pd^IV^. For example, in 2008, Inoue and coworkers demonstrated the PdCl_2_-catalysed C–H arylation of naphthalene with aryl stannanes.^12^ This reaction was selective for arylation at the α-position of naphthalene (α : β ratio = 3.5 : 1, Fig. 10a) and afforded modest 40% yield. A variety of other substrates were evaluated and phenanthrene was found to afford the best yield (80%) as well as high selectivity for the 9-position (Fig. 10b). A mechanism involving naphthalene or phenanthrene C–H activation at Pd^IV^ was proposed; however, minimal evidence is provided to support this pathway.

**Fig. 11:**
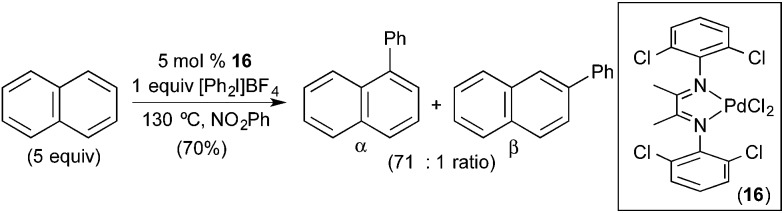
C–H arylation of naphthalene with [Ph_2_I]BF_4_ *via* proposed C–H activation at Pd^IV^.

More recently, our group demonstrated the C–H arylation of naphthalene using diaryliodonium salts as both the oxidant and aryl source (Fig. 11).^13^ In this system, the selectivity of C–H cleavage could be tuned through the appropriate selection of supporting ligand. Simple Pd salts, such as Pd(OAc)_2_ and PdCl_2_, afforded modest yields and selectivities for the C–C coupled products (yields ranging from 12–24% and α : β selectivities from 5 : 1 to 13 : 1). The yield and selectivity could be enhanced dramatically through the use of N–N chelating L type ligands, and the optimal diimine Pd catalyst (**16** in Fig. 11) afforded 70% yield and >70 : 1 selectivity for the α-arylated product. Notably, since this work, complementary β selectivity has been achieved in the same transformation by employing a platinum catalyst.^4*c*^

**Fig. 12:**
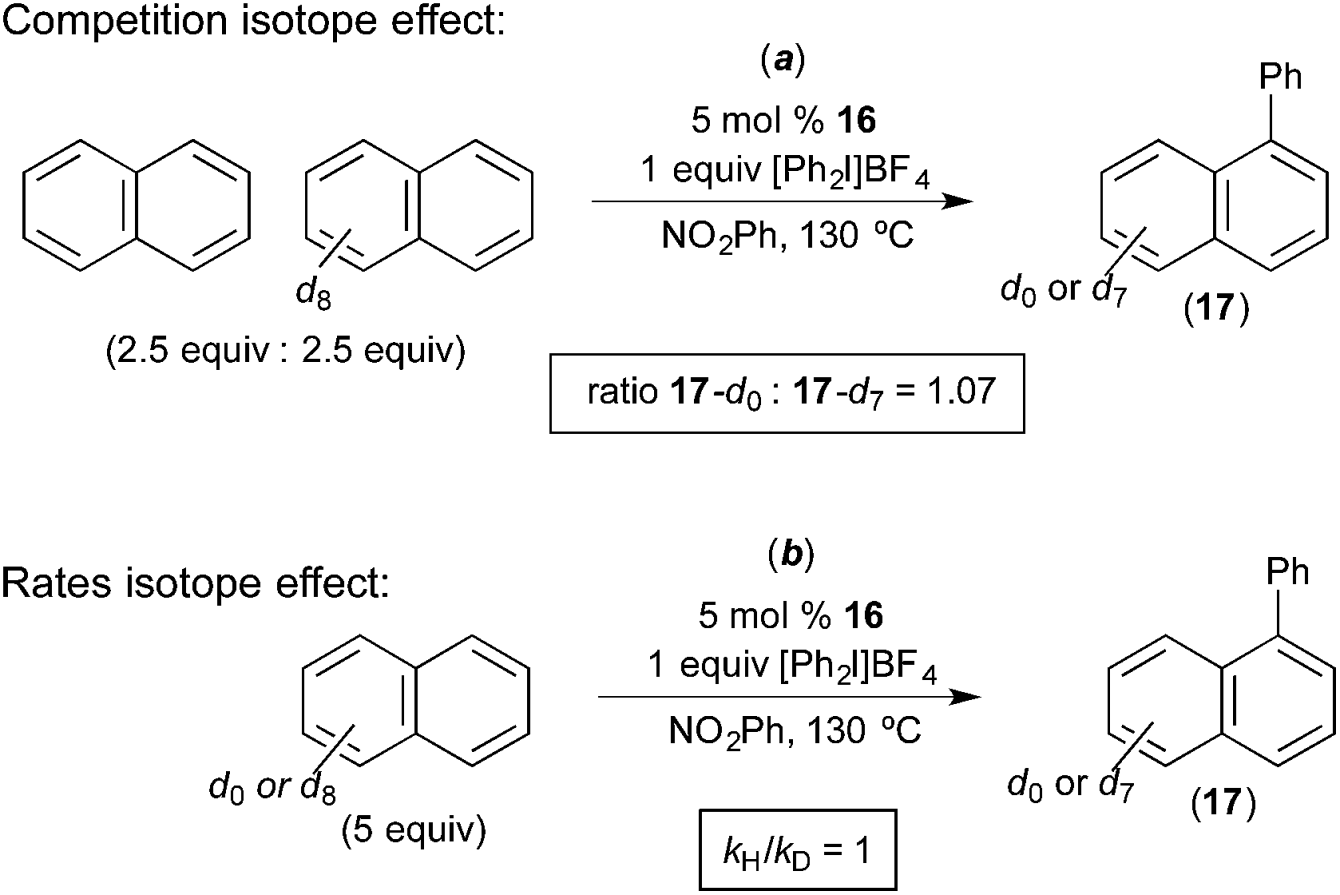
Isotope effect studies of naphthalene C–H arylation.

Rate studies of the Pd-catalyzed naphthalene arylation showed 1^st^ order kinetics in [Ar_2_I]^+^ and zero order dependence on [naphthalene]. Isotope effect studies were conducted using naphthalene and naphthalene-*d*_8_. The initial rate of the C–H arylation reaction was essentially identical with each of these two substrates (*k*_H_/*k*_D_ = 1, Fig. 12b). Furthermore, a competition between naphthalene and naphthalene-*d*_8_ afforded a product ratio corresponding to an H/D competition isotope effect of 1.08 (**17**-*d*_0_/**17**-*d*_7_ = 1.08, Fig. 12a). This is very similar to the results obtained by Michael in analogous competition experiments (Fig. 6). Naphthalene was found to be the best substrate for this reaction, and arenes without an extended π-system (*e.g.*, anisole, benzene, chlorobenzene, veratrole) afforded low yields and selectivities. On the basis of these investigations, the oxidation of the Pd^II^ catalyst by the aryliodonium salt was proposed to be the rate-determining step, and the C–H activation of naphthalene was proposed to occur at the resulting Pd^IV^ centre. Additionally, a two-step C–H activation mechanism analogous to that put forth by Michael (Fig. 7) was proposed in this system. The first step is proposed to involve π-coordination of the substrate to Pd^IV^ (a step that should be facilitated by the extended π-system of naphthalene) followed by subsequent C–H cleavage at the Pd^IV^ centre.

**Fig. 13:**
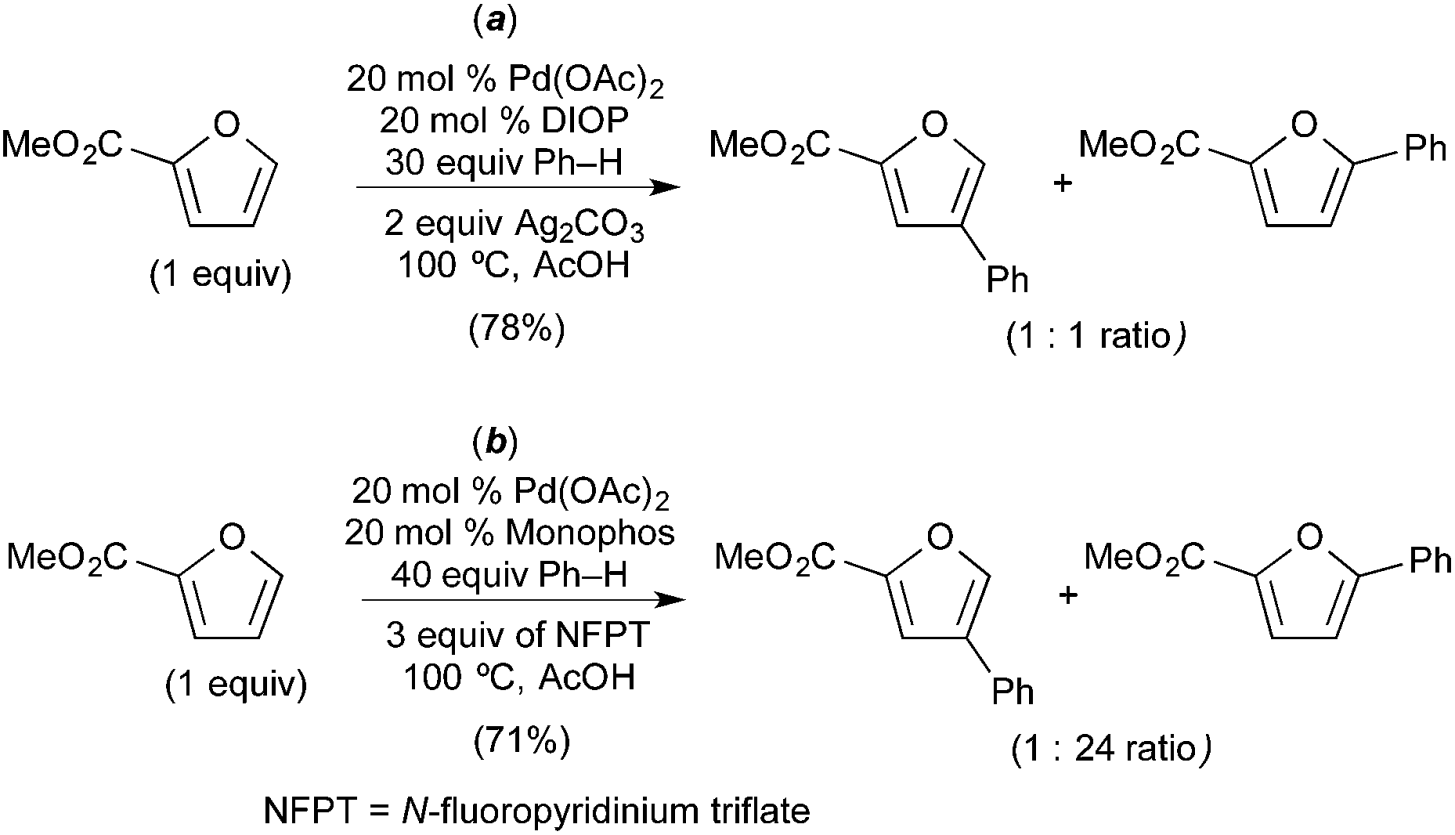
Oxidative coupling of furans and benzene: proposed furan C–H activation at Pd^II^ (a) and Pd^IV^ (b) depending on oxidant.

**Fig. 14:**
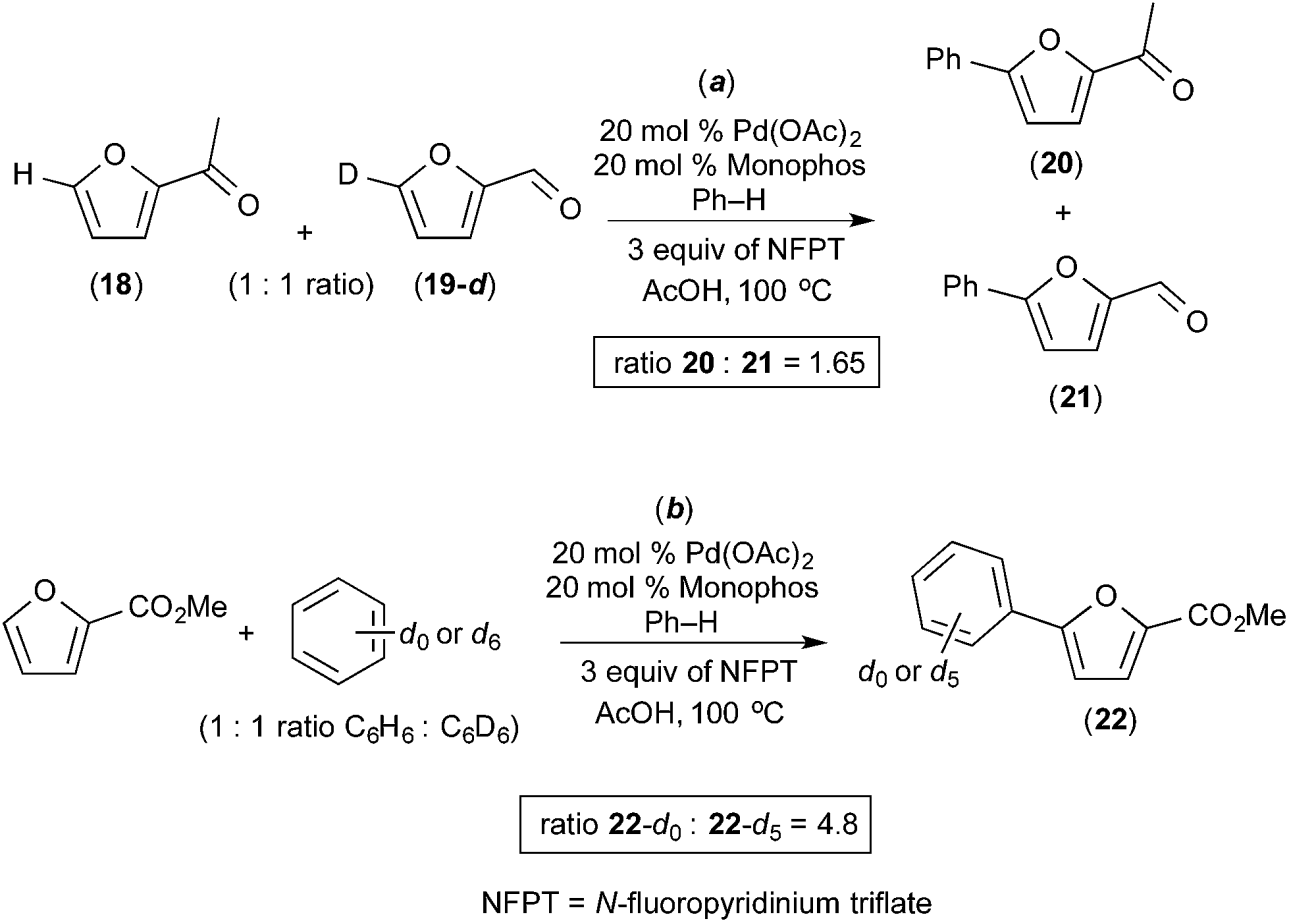
Isotope effect studies in benzene/furan oxidative coupling with NFPT as oxidant.

Seayad recently described the selective C–H/C–H oxidative coupling of furans with arenes (Fig. 13).^14^ In this system, the site selectivity of furan C–H activation could be modulated based appropriate selection of the terminal oxidant. Using AgCO_3_ as oxidant, the authors observed poorly selective activation of the furan (C-4/C-5 arylated products were formed in an ∼1 : 1 ratio, Fig. 13a). Notably, Ag_2_CO_3_ is unlikely to promote oxidation of Pd^II^ to Pd^IV^. In contrast, the use of *N*-fluoropyridinium triflate (NFTP), an “F^+^” oxidant that is well known to promote the oxidation of Pd^II^ to Pd^IV^,^15^ afforded >20 : 1 C-5 selectivity in most cases. In the NFTP system, large isotope effect (ratio of products **22**-*d*_0_/**22**-*d*_5_ = 4.8) was observed when the reaction was run as a competition between benzene and benzene-*d*_6_ (Fig. 14b). In contrast, the competition between furan **18** and deuterated furan **19-*d*** resulted in a relatively small quasi isotope effect of 1.7 (the substrates are slightly different, so this is not a true isotope effect; however, the authors state that the rate of arylation is similar for the two substrates) (Fig. 14a). The authors propose a mechanism initiated by initial oxidation of ligated Pd^II^ to Pd^IV^ by NFPT and subsequent C–H activation of the two substrates. An alternative possibility involving benzene activation at Pd^II^, oxidation with NFPT and subsequent furan activation at Pd^IV^ is also possible, and perhaps more likely based on the related reactions described above.

The examples described above summarize the current state of the art in catalytic transformations proposed to proceed *via* C–H activation at Pd^IV^. These examples show encouraging selectivity trends and demonstrate that valuable synthetic methods can be achieved with C–H activation at Pd^IV^ as a likely step. At this stage, most of these transformations have been discovered serendipitously rather than through reaction design. However, moving forward it would be important to rationally design catalytic sequences involving C–H activation at a Pd^IV^ centre. A uniting feature of the transformations discussed above is the use of strong oxidants, with “F^+^” reagents, hypervalent iodine reagents, and inorganic peroxides being particularly common choices. In addition, many these transformations are believed to involve the generation of a Pd^II^–C bond prior to oxidation of Pd^II^ to Pd^IV^. This likely serves to accelerate the oxidation event. Finally, most of the catalysts and intermediates in these transformations possess oxidatively stable ligands that are unlikely to participate in competing reductive elimination. All of these features should serve as key design considerations as new reactions are developed.

## Direct observation of C–H activation at Pd(iv)

**Fig. 15:**
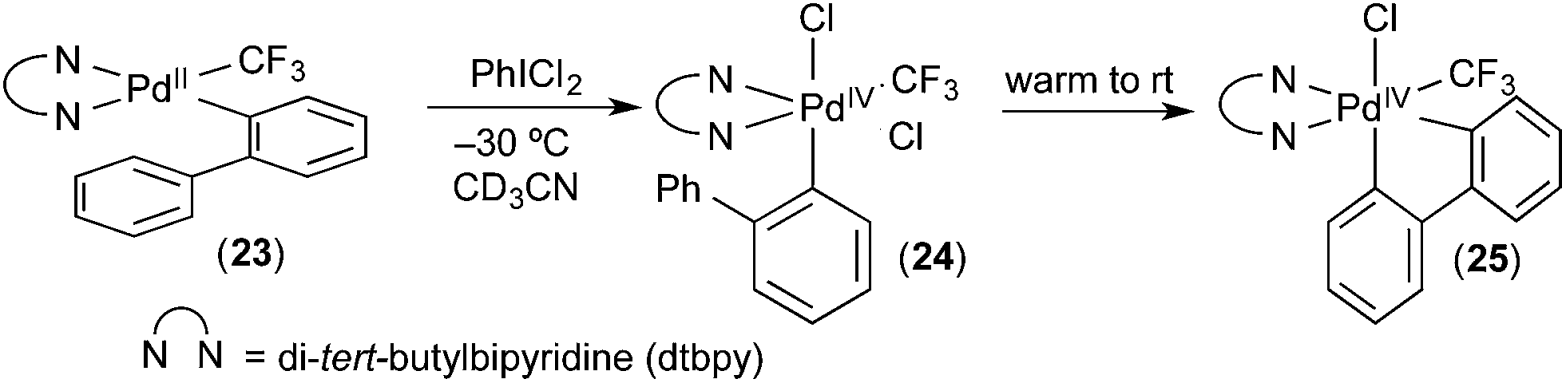
First direct observation of C–H activation at Pd^IV^.

Our group has developed organometallic model systems in order to directly observe and study this fundamental reaction. Such studies should ultimately assist in the rational design of new catalytic processes that incorporate this elementary step. The Pd^IV^ model complexes were carefully designed to accelerate C–H activation while slowing competing reductive elimination processes from Pd^IV^. These complexes were designed so that the C–H activation would be intramolecular. For example, in complex **24** (Fig. 15), studied by Racowski *et al.*,^16^ the biphenyl ligand was incorporated to enable intramolecular C–H activation, which is typically more facile than the corresponding intermolecular reactions. In addition, the CF_3_ ligand was included because aryl-CF_3_ reductive elimination is known to be relatively slow from Pd^IV^, particularly at low temperatures.^17^ Complex **24** was generated *in situ* by the oxidation of (bpy)Pd^II^(2-biphenyl) (CF_3_) complex **23** with PhICl_2_ at –30 °C. Warming complex **24** to room temperature resulted in intramolecular activation of the 2-aryl substituent to form the cyclometalated Pd^IV^ product **25**. To our knowledge, this was the first direct observation of C–H activation at a Pd^IV^ centre.

**Fig. 16:**
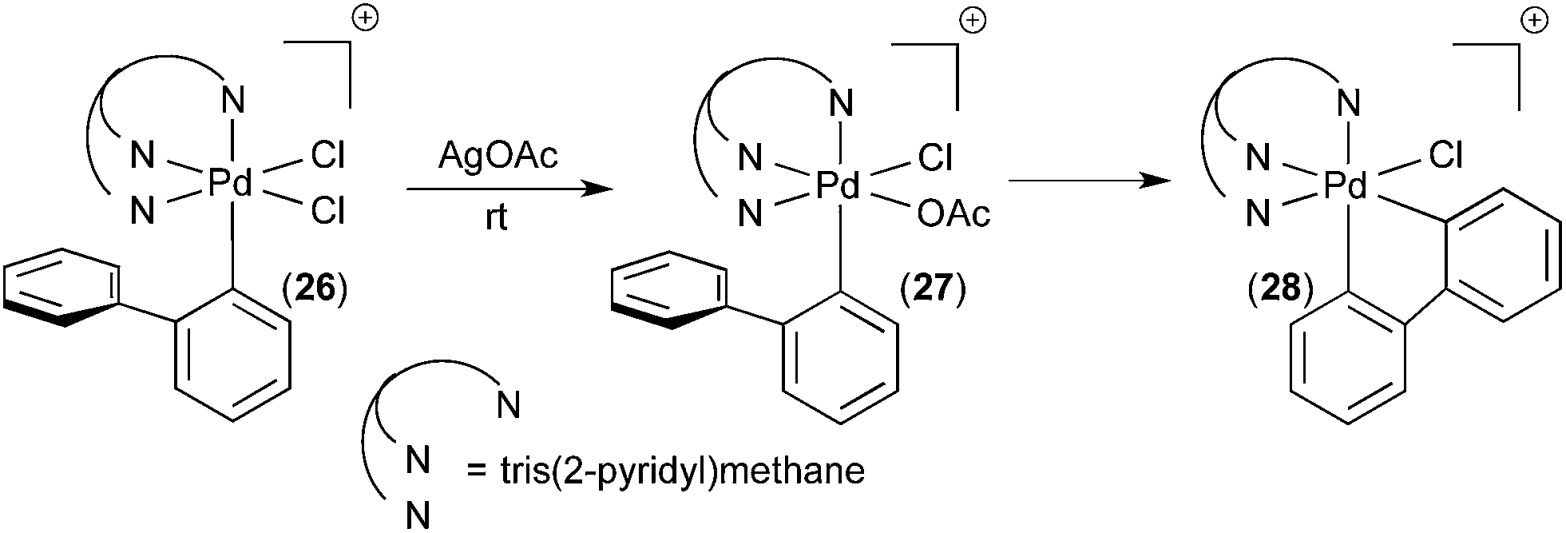
C–H activation at Pd^IV^ complex **27**.

In a follow up study, the related complex **26** (Fig. 16) was synthesized *via* oxidation of a Pd^II^ precursor with PhICl_2_.^18^ The tridentate tris(2-pyridyl)-methane ligand (Py_3_CH) was a key design feature in this study. This strongly coordinating tridentate ligand is well-known to stabilize octahedral Pd^IV^ species relative to analogues with bidentate nitrogen donors like bipyridine.^19^ Thus, it was anticipated that the Py_3_CH ligand would slow C–H activation and enable more detailed mechanistic investigations of this process. Indeed, the Pd^IV^ aryl complex **26** proved stable at room temperature and could be fully characterized by 1D and 2D NMR, HRMS, and X-ray crystallography. Complex **26** did not undergo C–H activation, even upon heating to 90 °C in CDCl_3_. Instead, C–Cl bond-forming reductive elimination was observed under these conditions. However, when one of the chloride ligands in **26** was exchanged for an acetate, the resulting intermediate **27** underwent clean cyclometalation at room temperature to yield **28**. This result suggests that C–H activation at Pd^IV^ in this system likely occurs *via* a concerted metalation-deprotonation mechanism, analogous to C–H activation at Pd^II^.^20^

**Fig. 17:**
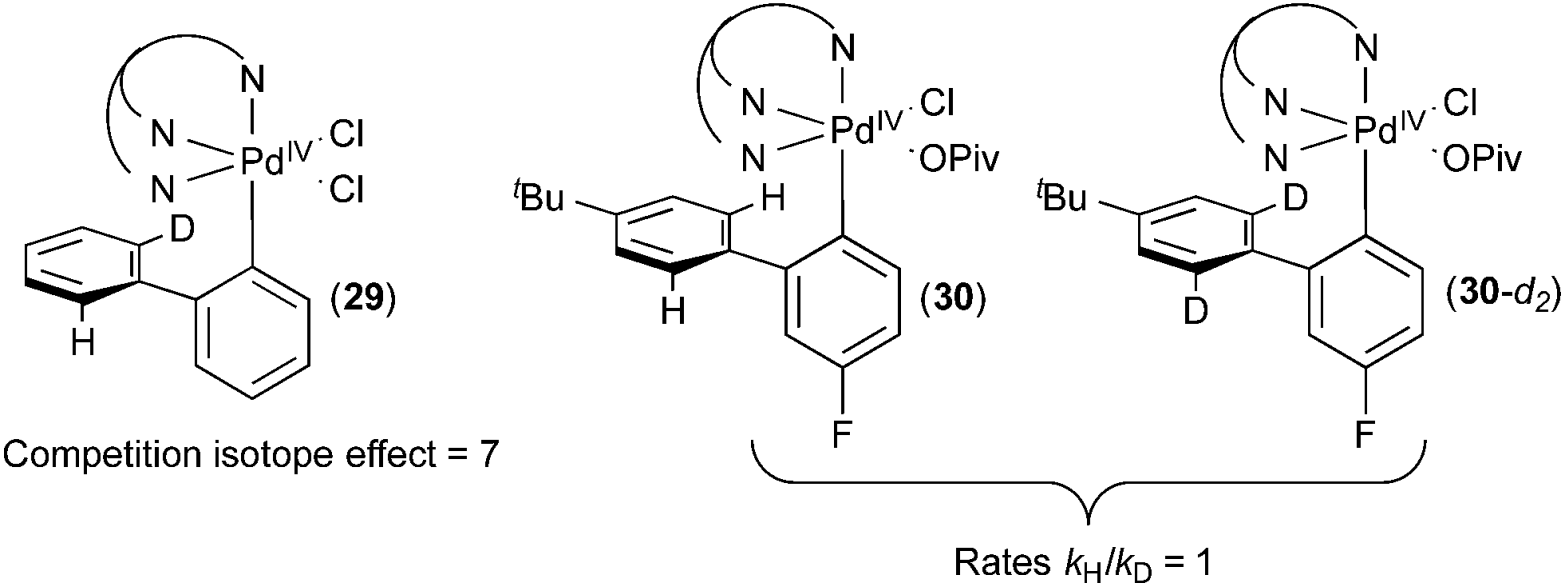
Isotope effects in C–H activation at Pd^IV^.

When complex **29** (Fig. 17) was treated with acetate, a product ratio consistent with an H/D competition isotope effect of 7 was obtained. In contrast, the initial rate of C–H activation at **30** *versus* **30**-*d*_2_ was essentially identical (*k*_H_/*k*_D_ = 1). These results in combination with a variety of studies of the dynamic behaviour of complex **26** indicate that C–H cleavage is not the rate determining step in the C–H activation process in this system. The similarity between the KIE observed on this system to those observed in catalytic systems further supports the mechanisms proposed for the catalytic reactions described above.

These two examples chronicle C–H activation at discrete and, in one case, isolable Pd^IV^ complexes. It is also noteworthy that C–H activation at the Pd^IV^ centre can be facile at room temperature or below with bidentate ligands or in the presence of acetate ion. Future work on isolated palladium complexes may explore an intermolecular C–H activation event at Pd^IV^.

## Conclusions

The catalytic functionalization of C–H bonds *via* high valent palladium is a powerful manifold for developing synthetically useful transformations. This mini-review has summarized experiments supporting the viability of C–H bond activation at Pd^IV^ and has described mechanistic studies of the C–H bond cleavage event. Synthetically useful catalytic cycles that utilize C–H activation at Pd^IV^ remain limited; however, there are great opportunities in this area due to the potential for unique selectivity in these transformations. Although the presently described work is mostly limited to catalytic C–H arylation sequences, as this method is more fully understood, one can anticipate its application to more diverse scaffolds and functionalizations.
